# Supplementary material for: Identification of photoperiod-induced specific miRNAs in the adrenal glands of Sunite sheep (Ovis aries)
Source: Front Vet Sci. 2022 Jul 22;9:888207. doi: 10.3389/fvets.2022.888207 (PMC9354845; doi:10.3389/fvets.2022.888207)
Supplement: Supplementary file 1 [file Data_Sheet_1.ZIP › Supplementary file/miRDeep2 file.zip!/miRDeep2 file/Novel_Structure/SP42A3/Novel_158.pdf]

Provisional ID	:	NC_019472.2_22064
Score total	:	1.2
Score for star read(s)	:	-1.3
Score for read counts	:	0
Score for mfe	:	0.9
Score for randfold	:	1.6
Score for cons. seed	:	
Total read count	:	13
Mature read count	:	7
Loop read count	:	0
Star read count	:	6

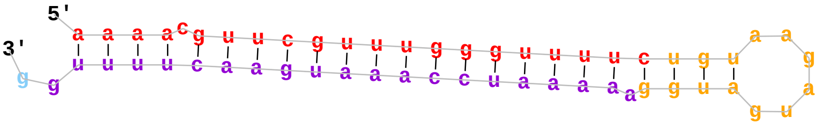

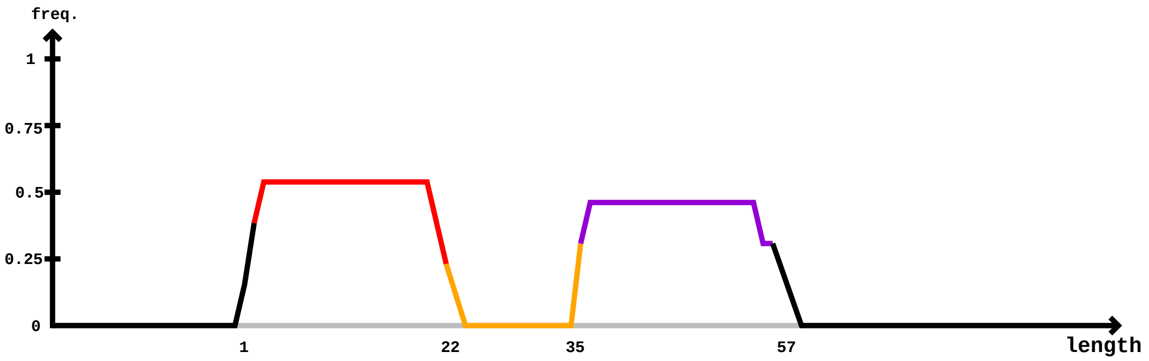


```
                    Mature                              Star
5'- gcaaaauauuggguagaucaaaacguucguuuggguuuucuguaagaugauggaaaaauccaaaugaacuuuugggccaacccauuaauaagcuauuucucuguacugu   -3'    obs
    gcaaaauauuggguagaucaaaacguucguuuggguuuucuguaagaugauggaaaaauccaaaugaacuuuugggccaacccauuaauaagcuauuucucuguacugu          exp
    ((....((.(((((.(.((((((.(((((((((((((((((((......)))).)))))))))))))))))))))..).))))).))....))................    reads      mm          sample
    ..................caaaaAguucguuuggguuu.......................................................................    2          1           seq
    ...................aaaaAguucguuuggguuuuc.....................................................................    3          1           seq
    ....................aaacguucguuuggguuuu......................................................................    2          0           seq
    .....................................................aaaaaCccaaaugaacuuu.....................................    1          1           seq
    .....................................................aaaaaCccaaaugaacuuuug...................................    2          1           seq
    .....................................................aaaaaCccaaaugaacuuuugg..................................    1          1           seq
    ......................................................aaaaCccaaaugaacuuu.....................................    1          1           seq
    ......................................................aaaauccaaaugaacuuuugg..................................    1          0           seq
```
